# Editorial: Adult stem cells for regenerative medicine: From cell fate to clinical applications

**DOI:** 10.3389/fcell.2022.1069665

**Published:** 2022-10-31

**Authors:** Jingting Li, Sudjit Luanpitpong, Pakpoom Kheolamai

**Affiliations:** ^1^ Institute of Precision Medicine, The First Affiliated Hospital, Sun Yat-Sen University, Guangzhou, China; ^2^ Department of Burns, The First Affiliated Hospital, Sun Yat-Sen University, Guangzhou, China; ^3^ Department of Dermatology, The First Affiliated Hospital, Sun Yat-Sen University, Guangzhou, China; ^4^ Department of Neurosurgery, Institute of Precision Medicine, The First Affiliated Hospital, Sun Yat-Sen University, Guangzhou, China; ^5^ Siriraj Center of Excellence for Stem Cell Research, Faculty of Medicine Siriraj Hospital, Mahidol University, Bangkok, Thailand; ^6^ Center of Excellence in Stem Cell Research and Innovation, Faculty of Medicine, Thammasat University, Bangkok, Thailand

**Keywords:** stem cells, regenerative medicine, mesenchymal stem cells, bone marrow mesenchymal stem cells, dental pulp stem cells, limbal stem cells, epidermal stem cells, skeletal stem cells

Adult stem cells have emerged as a key player in cell-based therapy in regenerative medicine. With the multilineage differentiation capability and ability to secrete various potent bioactive molecules, hematopoietic stem/progenitor cells (HSPCs) and mesenchymal stem cells (MSCs; also known as multipotent or mesenchymal stromal cells), in particular, have been considered a potential treatment for many debilitating diseases caused by injuries, inflammation, and age-related degeneration. The transplantation of these cells has been consistently shown to be safe; however, their therapeutic potential for many diseases and conditions varies between studies, attributable mainly to the differences in stem cell sources, donor selection, the isolation and expansion procedure, characterization criteria, number of transplanted cells, and disease severity. A major challenge when using adult stem cells for therapeutic applications includes the heterogeneous population of cells at the time of derivation and upon expansion in culture. The same type of adult stem cells derived from different tissues or even the same tissue from different donors exhibits distinct characteristics and biological properties, making the outcome of stem cell therapy difficult to be predicted. To improve the efficacy and reproducibility of stem cell therapy in regenerative medicine, a reliable characterization and a more thorough understanding of the mechanisms underlying the therapeutic effects of adult stem cells from various sources are critical. One of the well-established therapeutic mechanisms of HSPCs and MSCs involves the release of bioactive molecules, which have been shown to reduce inflammation, increase cell viability, and enhance tissue regeneration in injured tissues. In this issue, Norte-Muñoz et al. demonstrated the prosurvival effect of cytokines released from bone marrow-derived MSCs (BM-MSCs) on mouse retinal ganglion cells that enhanced axonal regeneration of the mouse optic nerve. The therapeutic effects of BM-MSCs largely depend on the immunocompatibility between donors and recipients, and hence, the type of transplant in which the higher compatibility is generated is the better outcome. A report by Li et al. also demonstrated a novel therapeutic mechanism of rapamycin, a drug used to treat immune-mediated bone marrow failure, by showing that the drug induces BM-MSCs to release granulocyte–macrophage colony-stimulating factor (GM-CSF), which promotes the expansion of myeloid-lineage cells while suppressing the subsequent differentiation of those myeloid cells to granulocytes in a mouse model.

Since the early days of stem cell therapy, HSPCs, MSCs, and endothelial progenitor cells (EPCs) have been used to treat various chronic diseases, especially diabetes and vascular complications associated with diabetes, such as myocardial infarction, ischemic stroke, and peripheral vascular disease. Two review articles by Yu et al. and Khodayari et al. provide an overview of the use of various adult stem cells to treat diabetic foot ulcers and limb ischemia, which are the leading causes of morbidity in diabetic patients. However, the significant hurdle to stem cell therapy for diabetes and its related diseases is the low survival rate of the transplanted cells, especially in the ischemic and inflammatory microenvironments of the target tissues. In this regard, Zhang et al. summarized the various types of programmed cell death that compromise the survivability of MSCs after transplantation and the potential strategies to prevent them. This insight is essential for developing an intervention that increases the overall survival of transplanted MSCs, especially in patients whose tissues experience chronic ischemic and inflammatory conditions, such as diabetes and atherosclerosis. Reconstruction of the bone and muscle is one of the most promising clinical applications of adult stem cells. To make the reconstruction processes more efficient, the optimization of biocompatibility scaffolds and a better understanding of the molecular events accompanying the differentiation of MSCs toward the desirable lineages are critical. A report by Xin et al. showed the potential of CHNQD-00603, a modified natural product derived from an anamorphic fungus *Scopulariopsis* sp., in inducing osteogenic differentiation of BM-MSCs by enhancing their autophagy. A review article by Shen and Shi also highlights recent advances in identifying the molecular mechanisms underlying the interaction between MSCs and their microenvironment during osteogenic differentiation that could be used to improve the culture conditions to induce bone formation. In addition to the bone marrow and their BM-MSCs, the periosteum has been shown to play essential roles in bone formation in physiological and pathological conditions. A review article by Zhang et al. summarized current knowledge on lesser-known periosteum-derived periosteal skeletal stem cells (P-SSCs) and their role in bone formation after injury. These cells might be used as an alternative source of MSCs for bone reconstruction in addition to BM-MSCs, which are very limited in supply. Another source of MSCs that shows great potential in tissue engineering is dental pulp stem cells (DPSCs), which can be used to regenerate a typically irreplaceable dental pulp in endodontic treatment. In their review article, Kwack and Lee summarized the recent updates and limitations in using DPSCs to regenerate dental pulp in the clinics.

Although the use of stem cells to generate skin and corneal tissue pieces for transplantation has been established for quite some time, the reconstruction of these tissues requires a large number of cells, which remains a significant hurdle. Therefore, an effective strategy for expanding epidermal and corneal cells while preserving their differentiation potential is critical. A report by Sun et al. uncovered the signaling pathways and several target genes involved in the long-term expansion of human epidermal cells by single-cell RNA sequencing and predicted the possibility of cell–cell communication using CellChat, while a commentary article by Ji et al. provided helpful information about the optimized culture conditions, e.g., by adding small molecular compounds, for expanding mouse limbal stem cells, a corneal precursor. This information could be beneficial in discovering candidate targets to induce the regeneration of the injured skin and corneal tissues in patients. A research article by Shi et al. also reported the establishment of human urine-derived stem cells (USCs), which have extensive expansion capacity while maintaining their multilineage differentiation. This alternative source of MSCs that could be harvested from every patient using a non-invasive procedure could potentially be used for autologous stem cell transplantation.

Terminally differentiated skeletal muscle contains a population of stem cells called satellite cells, but its ability to regenerate a new muscle fiber after an injury is limited. A report by Kim et al. showed that a specific population in the pharyngeal muscle called fibroadipogenic progenitors (FAPs) induced the proliferation of satellite cells by releasing hepatocyte growth factor (HGF). Such a finding provides valuable information regarding the interaction between various cell populations in the muscle that might be used to enhance skeletal muscle regeneration by inducing the proliferation of its satellite cells.

We hope the novel discovery and insight provided by these research and review articles will enhance our knowledge about the great potential of various adult stem cells in regenerative medicine and bring us closer to real therapeutic interventions in the near future.

